# [Retracted] Liraglutide repairs the infarcted heart: The role of the SIRT1/Parkin/mitophagy pathway

**DOI:** 10.3892/mmr.2024.13192

**Published:** 2024-03-04

**Authors:** Huiying Qiao, Haiyan Ren, He Du, Minfang Zhang, Xiaofang Xiong, Rong Lv

Mol Med Rep 17: 3722–3734, 2018; DOI: 10.3892/mmr.2018.8371

Following the publication of this paper, it was drawn to the Editors’ attention by a concerned reader that the immunofluorescence data shown in Fig. 2G, the mitochondria- and lysosome-stained images in Fig. 3C, the JC-1 staining images in Fig. 4C and the immunofluorescence data in Fig. 5G were strikingly similar to data appearing in different form in other articles written by different authors at different research institutes that had either already been published elsewhere prior to the submission of this paper to *Molecular Medicine Reports*, or were under consideration for publication at around the same time. In view of the fact that certain of the abovementioned data had already apparently been published previously, the Editor of *Molecular Medicine Reports* has decided that this paper should be retracted from the Journal. After having been in contact with the authors, they agreed with the decision to retract the paper. The Editor apologizes to the readership for any inconvenience caused.

